# Hippocampus-sparing radiotherapy using volumetric modulated arc therapy (VMAT) to the primary brain tumor: the result of dosimetric study and neurocognitive function assessment

**DOI:** 10.1186/s13014-018-0975-4

**Published:** 2018-02-20

**Authors:** Kyung Su Kim, Chan Woo Wee, Jin-Yong Seok, Joo Wan Hong, Jin-Beom Chung, Keun-Yong Eom, Jae-Sung Kim, Chae-Yong Kim, Young Ho Park, Yu Jung Kim, In Ah Kim

**Affiliations:** 1 Departments of Radiation Oncology, Seoul National University College of Medicine, Seoul, Republic of Korea; 2 Department of Radiation Oncology, Seoul National University Bundang Hospital, 166 Gumiro, Seongnamsi, Kyeonggido 463-707 South Korea; 3 Department of Neurosurgery, Seoul National University Bundang Hospital, Seongnamsi, Republic of Korea; 4 Department of Neurology, Seoul National University Bundang Hospital, Seongnamsi, Republic of Korea; 5 Department of Internal Medicine, Seoul National University Bundang Hospital, Seongnamsi, Republic of Korea

**Keywords:** Primary brain tumor, Brain radiotherapy, Volumetric modulated arc therapy, Hippocampus, Neurocognitive function test

## Abstract

**Background:**

We hypothesized that hippocampal-sparing radiotherapy via volumetric modulated arc therapy (VMAT) could preserve the neurocognitive function (NCF) of patients with primary brain tumors treated with radiotherapy.

**Methods:**

We reviewed data from patients with primary brain tumors who underwent hippocampal-sparing brain radiotherapy via VMAT between February 2014 and December 2015. The optimization criteria for the contralateral hippocampus was a maximum dose (D_max_) of less than 17 Gy. For NCF evaluations, the Seoul Verbal Learning Test for total recall, delayed recall, and recognition (SVLT-TR, DR, and Recognition) was performed at baseline and at seven months after radiotherapy.

**Results:**

A total of 26 patients underwent NCF testing seven months after radiotherapy. Their median age was 49.5 years (range 26–77 years), and 14 (53.8%) had grade III/IV tumors. The median D_max_ to the contralateral hippocampus was 16.4 Gy (range 3.5-63.4). The median mean dose to the contralateral hippocampus, expressed as equivalent to a 2-Gy dose (EQD_2/2_), was 7.4 Gy_2_ (0.7–13.1). The mean relative changes in SVLT-TR, SVLT-DR, and SVLT-Recognition at seven months compared to the baseline were − 7.7% (95% confidence interval [CI], − 19.6% to 4.2%), − 9.2% (95% CI, − 25.4% to 7.0%), and − 3.4% (− 12.7% to 5.8%), respectively. Two patients (7.7%) showed deteriorated NCF in the SVLT-TR and SVLT-DR, and three (11.5%) in the SVLT-Recognition. The mean dose of the left hippocampus and bilateral hippocampi were significantly higher in patients showing deterioration of the SVLT-TR and SVLT-Recognition than in those without deterioration.

**Conclusions:**

The contralateral hippocampus could be effectively spared in patients with primary brain tumor via VMAT to preserve the verbal memory function. Further investigation is needed to identify those patients who will most benefit from hippocampal-sparing radiotherapy of the primary brain tumor.

**Electronic supplementary material:**

The online version of this article (10.1186/s13014-018-0975-4) contains supplementary material, which is available to authorized users.

## Background

Radiotherapy is an integral part of brain cancer treatment. It improves the progression-free survival (PFS) of patients with low-grade glioma and is also a standard treatment after surgery with or without chemotherapy in cases of high-grade glioma.

While the tumor itself may affect the neurocognitive function (NCF) of patients, radiotherapy is also associated with declined NCF. In particular, due to the association between the hippocampal neural stem and memory function, radiation therapy of the hippocampal area is associated with deteriorated cognitive and memory functions [1–3].

Effective hippocampal sparing was made possible with the development of sophisticated radiotherapy delivering techniques such as intensity modulated radiotherapy (IMRT) [2, 4, 5]. Hippocampal-sparing whole brain radiotherapy (WBRT) for brain metastases was proven to be effective in a recent clinical trial. The Radiation Therapy Oncology Group (RTOG) 0933 trial enrolled 113 patients with brain metastases treated with hippocampal-sparing WBRT, showing promising results in the preservation of memory function, compared to historical data [6].

However, unlike WBRT, the hippocampal-sparing strategy for the radiotherapy treatment of primary brain tumor has not been thoroughly evaluated. Although the dosimetric feasibility has been reported in a number studies [2, 7–17], to our knowledge, there has been no report on the association between NCF and hippocampal-sparing radiotherapy.

Therefore, we report a dosimetric profile of hippocampal-sparing radiotherapy for the treatment of primary brain tumor as well as the change in NCF of the patients.

## Methods

### Patient Selection

Hippocampal-sparing radiotherapy to the brain was delivered using the volumetric modulated arc therapy (VMAT) technique between February 2014 and December 2015 at Seoul National University Bundang Hospital. A total of 74 patients have received partial brain irradiation for primary brain tumor, 69 of whom agreed to undergo NCF testing at baseline. Among them, 26 patients also underwent NCF testing 7 months after radiotherapy. After obtaining approval from the Institutional Review Board (No. B-1411/276-105), we analyzed the medical records and dosimetric parameters of these patients.

### Radiotherapy Simulation

All patients were positioned using a Variable Axis Baseplate ™ (CIVCO Medical Instruments, Kalona, IA, USA). The head was inclined as previously described [18]. The computed tomography (CT) scans were acquired by using a Brilliance CT Big Bore™ CT simulator (Philips, Cleveland, OH, USA) with a slice thickness of 2 mm.

### VMAT Plan Technique

All CT images of the patients were fused with their recent magnetic resonance (MR) images. The hippocampus was delineated according to RTOG guidelines [19]. All contours were delineated by the same radiation oncologist (I.A.K) and each delineation was peer-reviewed by K.S.K and J.Y.S. An optimization criterion for the hippocampus was a maximum dose (D_max_) of less than 17 Gy. However, we did not compromise the coverage of the planning target volume (PTV). In cases where the ipsilateral hippocampus was close to the PTV, we tried to meet the dosimetric criteria for the contralateral hippocampus. The brain stem, optic chiasm, and optic apparatus were also delineated. The other organs at risk were prioritized over the hippocampal dose constraint.

For primary brain tumors, the clinical target volume (CTV) was calculated with an adequate margin of 1.5 – 2.0 cm from the tumor bed or gross tumor. The prescription dose was 60 Gy to the PTV for high-grade glioma and 40 – 56 Gy for low-grade glioma. The mean dose (D_mean_) hippocampus was calculated as equivalent to a 2-Gy dose (EQD_2/2_) with α/β = 2.

### NCF

NCF was assessed using the Mini-Mental State Examination (MMSE); Seoul Verbal Learning Test (SVLT); and Rey Complex Figure Test, and Recognition Trial (RCFT) [20–23]. The SVLT is used to assess the verbal memory system using a list of 12 nouns with four words drawn from each of three semantic categories. The total recall (SVLT-TR) trial is the sum of the three learning trials. The SVLT also includes a 20-min delayed recall trial (SVLT-DR) and a yes/no delayed recognition trial (SVLT-Recognition). This last trial consists of a randomized list of 12 target words and 12 non-target words, six of which are drawn from the same categories as those of the targets. This study was standardized and norms that have been adjusted for age, education, and gender were developed for the elderly Korean population [22]. The NCF test was conducted at baseline and 7 months after radiotherapy. The relative differences were measured as ΔNCF = (NCF_B_-NCF_F_)/NCF_B,_ where B = baseline, F = follow-up, and the deterioration in the NCF test from baseline was defined as a z-score drop of 1.5 (drop of 1.5 standard deviations).

### Statistical analysis

The doses administered to the bilateral hippocampi and right and left hippocampus of the two groups were compared using Student’s t-tests. *P*-values less than 0.05 were considered to indicate statistically significant differences. Analyses were performed using PASW Statistics for Windows, Version 18.0 (SPSS Inc., Chicago, IL).

## Results

### Patients’ Characteristics

Of the 69 patients who agreed to undergo NCF testing at baseline, 26 also underwent the test 7 months after radiotherapy. Their median age was 49.5 years (range 26-77 years) and 57.7% of the patients were female. Twelve patients (46.2%) had WHO grade I or II tumor, whereas 14 patients (53.8%) had grade III or IV tumor. The median PTV volume was 173.1 cm^3^ (range 30.3-493.6) and the median prescribed dose was 60 Gy (range 40-60). Concurrent chemotherapy was administered to eight patients (30.8%) diagnosed with glioblastoma (Table 1).

**Table 1:** Patients’ and tumor characteristics

	Number		Percent
Median Age (range)	49.5 yrs. (26-77)		
Sex			
Male	11		42.3
Female	15		57.7
Diagnosis			
WHO I			
Pituitary adenoma	2	4	15.4
Megingioma	1		
Craniopharyngioma	1		
WHO II			
Oligodendroglioma	3	8	30.8
Atypical meningioma	3		
Diffuse astrocytoma	1		
Ependymoma	1		
WHO III			
Anaplastic astrocytoma	2	6	23.1
Anaplastic oligodendroglioma	2		
Anaplastic meningioma	1		
Anaplastic hemangiopericytoma	1		
WHO IV			
Glioblastoma	7	8	30.8
Gliosarcoma	1		
Surgery			
GTR/STR	22		84.6
Bx only/No surgery	4		15.4
Concurrent Chemotherapy	8		30.8
Location			
Frontal	5		19.2
Parietal	5		19.2
Temporal	6		23.1
Ocippital	1		3.8
Temporo-parietal	2		7.7
Fronto-temporal	1		3.8
Fronto-Prietal	1		3.8
Central	5		19.2
Planning Target Volume (PTV)			
Vol(cc)		173.1 cm^3^ (30.3-493.6)	
Prescribed dose		60 Gy (40-60)	

*GTR* gross total resection; STR, subtotal resection

### Dosimetric Analysis

The median doses to 100% of the structure (D_100%_) and D_max_ of the contralateral hippocampus were 7.2 Gy (range 0.6–11.7) and 16.4 Gy (range 3.5–63.4), respectively. The median D_mean_ expressed in EQD_2/2_ to the contralateral hippocampus was 7.4 Gy_2_ (range 0.7–13.1). The ipsilateral hippocampus received a higher dose. In addition, the median D_max_ and D_mean_ (EQD_2/2_) of the ipsilateral hippocampus were 40.9 Gy (range 5.7–64.3) and 10.3 Gy_2_ (range 1.0–62.3), respectively. The median values of the maximal doses to the brain stem and optic chiasm were 43.3 Gy (range 0.2–61.5) and 42.5 Gy (range 1.0–57.8), respectively. The other organs at risk could be effectively spared (Table 2).

**Table 2:** Dosimetric analysis

Dosimetric parameters	Median (range)
Hippocampus	
Contralateral	
Vol(cc)	1.8 cm^3^ (0.9-2.4)
D_100%_	7.2Gy (0.6-11.7)
D_max_	16.4 Gy (3.5-63.4)
D_mean_	12.3 Gy (1.3-19.7)
D_mean_ (EQD_2/2_)	7.4 Gy_2_ (0.7-13.1)
Ipsilateral	
Vol(cc)	1.7 cm^3^ (0.6-2.3)
D_100%_	8.4 Gy (0.7-60.0)
D_max_	40.9 Gy (5.7-64.3)
D_mean_	15.9 Gy (2.0-60.3)
D_mean_ (EQD_2/2_)	10.3 Gy_2_ (1.0-63.4)
Bilateral	
D_mean_	13.4 (1.8-38.3)
D_mean_ (EQD_2/2_)	8.3 Gy_2_ (0.9-31.3)
Optic nerve	
D_max_	31.7 Gy (0.5-58.5)
Optic chiasm	
D_max_	42.5 Gy (1.0-57.8)
Brain stem	
D_max_	43.3 Gy (0.2-61.5)
Eyeballs	
D_max_	13.2Gy (0.5-38.1)
Lenses	
D_max_	3.5Gy (0.4-14.0)

*D*_*100%*_ dose to 100% volume of the structure, *D*_*max*_ maximum dose, *D*_*mean*_ mean dose, *EQD*_*2/2*_ equivalent 2 Gy dose with α/β = 2

### NCF Test Results

Of the 26 patients who underwent neurocognitive testing at 7 months, two patients diagnosed with gliosarcoma and glioblastoma had progressive disease before 7 months. The other 24 patients presented with stable disease at 7 months. At the median follow-up of 13.9 months (range 7.0–25.6), the median PFS and overall survival were not reached. At the last follow-up, eight patients had progressed and one patient had died.

**Fig. 1:**
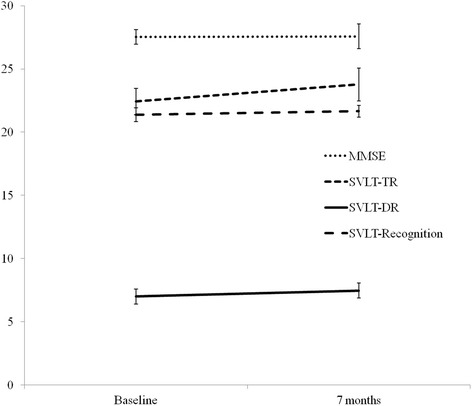
Mini-Mental State Examination (MMSE) and Seoul Verbal Learning Test (SVLT) score at baseline and at seven months

The NCF test results at baseline and at 7 months for the 26 patients are listed in Table 3 and Fig. 1. The mean relative change of SVLT-TR, SVLT-DR, and SVLT-Recognition at 7 months compared to the baseline were − 7.7% (95% confidence interval [CI], − 19.6% to 4.2%), − 9.2% (95% CI, − 25.4% to 7.0%, after excluding one patient with 0 at baseline), and − 3.4% (− 12.7% to 5.8%), respectively. The patients with deterioration in the tests included two (7.7%) in the SVLT-TR and SVLT-DR and three (11.5%) in the SVLT-Recognition. In regard to the RCFT, 24%, 8%, 8%, and 12% of the patients showed deterioration, respectively.

**Table 3:** Change of neurocognitive function (NCF) test at seven months from baseline

	Mean change from Baseline (%)^a^	95% CI	Probability of deterioration^b^(%)
MMSE	− 1.2	− 9.8 to 7.4	3.8
SVLT-Total recall	−7.7	−19.6 to 4.2	7.7
SVLT-Delayed recall	−9.2^c^	− 25.4 to 7.0^c^	7.7
SVLT-Recognition	−3.4	−12.7 to 5.8	11.5
RCFT-COPY	1.8	−7.4 to 11.1	24
RCFT-Immediate recall	−8.1	−36.2 to 20.0	8
RCFT-Delayed recall	−25.2^d^	− 52.8 to 2.5^d^	8
RCFT-Recognition	−3.8	−11.2 to 4.0	12

*MMSE* Mini-Mental State Examination, *SVLT* Seoul Verbal Learning Test, *RCFT* Rey Complex Figure Test and Recognition Trial

^a^ΔNCF = (NCF_B_-NCF_F_)/NCF_B,_ Where B = baseline and F = follow-up, (Minus change indicate improved NCF)

^b^Deterioration in NCF test from baseline defined as drop of z-score 1.5 (drop of 1.5 standard deviation)

^c^Exclusion of one patient with 0 test result at baseline

^d^Exclusion of one patient who could not be assessed at baseline

### Hippocampal Dose and Neurocognitive Impairment

We compared the hippocampal dose of the patients with varying NCF test results (Table 4 and Additional file 1: Table S1). We compared the right, left, contralateral, ipsilateral, and bilateral hippocampi mean doses (EQD_2/2_), respectively. The mean doses of the left hippocampus and bilateral hippocampi were significantly higher in patients with deterioration of SVLT-TR and SVLT-Recognition than in those without deterioration. The bilateral hippocampal mean dose was significantly higher in patients with impaired RCFT-Recognition test results (*p* = 0.042).

**Table 4:** Association between hippocampus dose and neurocognitive test deterioration

	Mean dose (EQD_2/2_)					
	Bilateral hippocampi	*P* value	Right hippocampus	P value	Left hippocampus	P value
SVLT-Total recall		0.033^a^		0.398		0.013^a^
No Deterioration (*n* = 23)	10.6 ± 6.5		15.7 ± 16.8		11.8 ± 14.1	
Deterioration (*n* = 3)	20.3 ± 11.5		7.2 ± 1.7		37.7 ± 27.6	
SVLT-Delayed recall		0.115		0.558		0.074
No Deterioration (*n* = 24)	11.0 ± 6.7		15.2 ± 16.6		13.1 ± 15.0	
Deterioration (n = 2)	19.8 ± 16.3		8.2 ± 0.1		36.0 ± 38.9	
SVLT-Recognition		0.003^a^		0.427		0.001^a^
No Deterioration (*n* = 23)	10.2 ± 6.0		13.8 ± 15.8		11.1 ± 11.7	
Deterioration (n = 3)	23.3 ± 9.4		21.8 ± 19.5		43.1 ± 30.9	
RCFT-COPY		0.469		0.211		0.261
No Deterioration (*n* = 20)	11.1 ± 6.6		16.9 ± 17.7		12.7 ± 14.9	
Deterioration (*n* = 6)	13.7 ± 10.7		7.5 ± 3.7		22.0 ± 24.6	
RCFT-Immediate recall		0.156		0.513		0.081
No Deterioration (n = 24)	11.1 ± 6.6		15.3 ± 16.5		13.1 ± 15.0	
Deterioration (*n* = 2)	19.0 ± 17.4		7.4 ± 1.2		36.0 ± 39.4	
RCFT-Delayed recall		0.156		0.513		0.081
No Deterioration (*n* = 24)	11.1 ± 6.6		15.3 ± 16.5		13.1 ± 15.0	
Deterioration (*n* = 2)	19.0 ± 17.4		7.4 ± 1.2		35.6 ± 39.4	
RCFT-Recognition		0.042^a^		0.406		0.257
No Deterioration (*n* = 23)	10.6 ± 6.5		13.8 ± 16.0		13.4 ± 15.2	
Deterioration (*n* = 3)	19.9 ± 11.5		22.1 ± 20.9		25.8 ± 32.7	

Numbers are represented as mean ± SD

*SVLT* Seoul Verbal Learning Test, *RCFT* Rey Complex Figure Test and Recognition Trial

^a^indicate statistical significance by student’s t-test

## Discussion

Numerous studies have assessed the association between the radiation dose to the hippocampus and memory function in patients [24, 25]. Furthermore, the NCF decline in patients treated with WBRT is associated with the hippocampal radiation dose [26, 27]. The recent development of radiotherapy techniques has made hippocampal-sparing radiotherapy possible, which was shown to be efficient in the WBRT in a recent clinical trial [6].

However, there are several considerations when applying the hippocampal-sparing strategy to primary brain tumors. First, compromising the target volume for hippocampal-sparing is not recommended. When treating brain metastases, hippocampal-sparing WBRT has an acceptable risk. Ghia et al. reviewed 100 patients with brain metastasis, reporting that 8% had metastases within 5 mm of the hippocampus [28]. The modest increase in the risk of recurrence could be balanced with salvage stereotactic radiosurgery. However, in primary brain tumor, the safety of compromising the target volume for the hippocampus has not been validated. In high-grade glioma, recurrences are most often located within 2 cm of the original tumor [29]. Moreover, the report that patients with glioblastoma involving the subventricular zone have decreased overall survival and PFS remains controversial [30, 31]. The recently published American Society for Radiation Oncology (ASTRO) guidelines for glioblastoma noted that given the absence of published data for the hippocampal-sparing in glioblastoma patients, the panel does not recommend compromising the target coverage for hippocampus protection [32].

Second, the hippocampi have a bilateral structure. In case the ipsilateral hippocampus is close to the target volume, we could at least spare the contralateral hippocampus by using the IMRT technique [8]. However, it is uncertain if this strategy could be beneficial for the preservation of the memory function. Lesion studies indicate that the left and right temporomesial structures are essential for verbal and visuospatial memory, respectively [33, 34]. Patients with left lobe-origin complex partial seizures have abnormalities in verbal memory [35], while those with nondominant foci may have deficits in visuospatial memory, even though this is less established [34]. Jalali et al. reported that radiotherapy doses to the left temporal lobe are predictors of neurocognitive decline [24]. In the current study, we could spare the contralateral hippocampus to the median value of D_mean_ (EQD_2/2_) to 7.4 Gy_2_. Moreover, the left hippocampal dose was significantly associated with SVLT, whereas the right hippocampal dose was not. In regard to the preservation of the verbal memory function, sparing the contralateral hippocampus with the right lobe lesion could be effective. In the current study, the patients had undergone RCFT, which evaluates visuospatial memory. However, we did not observe an association between the deterioration of RCFT results and the radiation dose to the right hippocampus. Further investigation to identify the association between the visuospatial memory function impairment and the radiation dose to the right hippocampus is required.

Third, unlike the WBRT, the target region differs among patients undergoing radiotherapy of the primary brain tumor. Therefore, comparisons of the hippocampal dosimetric profile and NCF toxicity are difficult. Several studies reported consistent results with those of our study regarding the dosimetric profile of the hippocampus when applying the hippocampal-sparing strategy using various IMRT techniques for the radiotherapy of the primary brain tumor [2, 7–17]. Pinkham et al. reported the dosimetric feasibility of hippocampal-sparing IMRT in grade II and grade III gliomas. They reported a median mean dose to the contralateral hippocampus of 24.9 Gy (range 5.1–58 Gy) [9]. Marsh and colleagues achieved mean doses of 15.8 Gy and 12 Gy for patients with high-grade and low-grade gliomas, respectively [13]. In regard to other critical structures, we achieved acceptable radiation doses for all vital organs.

The memory function deterioration is reportedly 30%–60% eight to 18 months after cranial irradiation for primary brain tumor [36–39]. In the RTOG 0933 trial, the probability of deterioration of the Hopkins Verbal Learning Test-Revised Delayed Recall score of patients who underwent hippocampal sparing radiotherapy was 17.2% at 6 months [6]. In the current study, the deterioration in the SVLT-DR test was 7.7%. However, direct comparison of this result with those of other studies has limitations. We only analyzed patients who underwent neurocognitive function tests at 7 months; the compliance with this test at 7 months was 38%, whereas the compliance of the NCF test at 6 months in the RTOG 0933 trial was 54%. Second, this study included patients with heterogeneous histology. Rapid progression of WHO IV disease might affect the neurocognitive function test. Of the two patients who progressed before the NCF test at 7 months, one patient with a left hippocampus dose as high as 63.4 Gy EQD_2/2_ exhibited an NCF test decline. Meanwhile, in patients with less aggressive histology, hippocampus-sparing radiotherapy may be more beneficial. However, the association between the integral dose to normal brain tissue and long-term neurocognitive changes should be carefully investigated in low-grade tumors especially in young patients. Further prospective studies with homogenous disease would clarify the benefit of hippocampal-sparing partial brain irradiation.

## Conclusion

We used VMAT to apply hippocampal-sparing radiotherapy to primary brain tumors. The hippocampus could be reasonably spared and NCF tests performed 7 months after radiotherapy showed promising results in the preservation of verbal memory function. The left hippocampal mean dose was associated with the deterioration of the memory function, while the right hippocampal mean dose was not. Further investigation is needed in order to select patients who will most benefit from hippocampal-sparing radiotherapy of the primary brain tumor.

## Additional file

Additional file 1: **Table S1.** Association between hippocampus dose and neurocognitive test deterioration. (DOCX 20 kb)

## Abbreviations

CT V: Clinical target volume
IMRT: Intensity modulated radiotherapy
MMSE: Mini-Mental State Examination
NCF: Neurocognitive function
PFS: Progression-free survival
PTV: Planning target volume
RCFT: Rey Complex Figure Test, and Recognition Trial
RTOG: Radiation Therapy Oncology Group
SVLT-TR,-DR-Recognition: Seoul Verbal Learning Test - total recall, delayed recall, recognition
VMAT: Volumetric modulated arc therapy
WBRT: Whole brain radiotherapy
