# Supplementary material for: Hippocampus-sparing radiotherapy using volumetric modulated arc therapy (VMAT) to the primary brain tumor: the result of dosimetric study and neurocognitive function assessment
Source: Radiat Oncol. 2018 Feb 20;13:29. doi: 10.1186/s13014-018-0975-4 (PMC5819694; doi:10.1186/s13014-018-0975-4)
Supplement: Supplementary file 1 — Table S1. Association between hippocampus dose and neurocognitive test deterioration. (DOCX 20 kb) [file 13014_2018_975_MOESM1_ESM.docx]

Supplementary Table 1. Association between hippocampus dose and neurocognitive test deterioration

|  | | Laterality of tumors | | Mean dose (EQD_2/2_) | | | |
| --- | --- | --- | --- | --- | --- | --- | --- |
|  | | Rt side tumor | Lt side tumor | Contralateral  hippocampus | P value | ipsilateral hippocampus | P value |
| SVLT-Total recall | |  |  |  | 0.927 |  | 0.183 |
|  | No Deterioration (n=23) | 13 | 10 | 7.05±2.9 |  | 20.5±19.6 |  |
|  | Deterioration (n=3) | 0 | 3 | 7.2±1.7 |  | 37.7±27.6 |  |
| SVLT-Delayed recall | |  |  |  | 0.566 |  | 0.35 |
|  | No Deterioration (n=24) | 13 | 11 | 7.0±2.9 |  | 21.4±20.6 |  |
|  | Deterioration (n=2) | 0 | 2 | 8.2±0.1 |  | 36.0±38.9 |  |
| SVLT-Recognition | |  |  |  | 0.093 |  | 0.002^*^ |
|  | No Deterioration (n=23) | 12 | 11 | 6.7±2.6 |  | 18.2±17.3 |  |
|  | Deterioration (n=3) | 1 | 2 | 9.6±3.0 |  | 55.3±10.0 |  |
| RCFT-COPY | |  |  |  | 0.630 |  | 0.960 |
|  | No Deterioration (n=20) | 11 | 9 | 7.2±2.9 |  | 22.4±20.4 |  |
|  | Deterioration (n=6) | 2 | 4 | 6.6±2.3 |  | 22.9±24.1 |  |
| RCFT-Immediate recall | |  |  |  | 0.851 |  | 0.365 |
|  | No Deterioration (n=24) | 13 | 11 | 7.0±2.9 |  | 21.4±19.6 |  |
|  | Deterioration (n=2) | 0 | 2 | 7.4±1.2 |  | 36.0±39.4 |  |
| RCFT-Delayed recall | |  |  |  | 0.851 |  | 0.365 |
|  | No Deterioration (n=24) | 13 | 11 | 7.0±2.9 |  | 21.4±19.6 |  |
|  | Deterioration (n=2) | 0 | 2 | 7.4±1.2 |  | 35.6±39.4 |  |
| RCFT-Recognition | |  |  |  | 0.844 |  | 0.111 |
|  | No Deterioration (n=23) | 11 | 12 | 7.0±2.9 |  | 20.1±19.4 |  |
|  | Deterioration (n=3) | 2 | 1 | 7.4±1.9 |  | 40.5±26.2 |  |

Numbers are represented as mean±SD

SVLT, Seoul Verbal Learning Test; RCFT, Rey Complex Figure Test and Recognition Trial  ^*^ indicate statistical significance by student’s t-test
